# Selective bronchial occlusion for the prevention of pneumothorax after transbronchial lung cryobiopsy in a pulmonary alveolar proteinosis patient: a case report

**DOI:** 10.3389/fmed.2023.1265373

**Published:** 2023-11-27

**Authors:** Hua-Man Wu, You-Li Wen, Xiao-Yu He, Zhi-Ping Deng

**Affiliations:** Department of Respiratory and Critical Care Medicine, Zigong First People’s Hospital, Zigong, Sichuan, China

**Keywords:** pulmonary alveolar proteinosis, transbronchial lung cryobiopsy, selective bronchial occlusion, diagnosis, case report

## Abstract

The diagnosis of pulmonary alveolar proteinosis (PAP) is based on biopsies. Compared with other methods of taking biopsies, transbronchial lung cryobiopsy (TBLC) has a higher diagnostic rate and the likelihood of pneumothorax. Selective bronchial occlusion (SBO) is an effective technique for treating intractable pneumothorax. However, there are no data available about SBO for the prevention of pneumothorax after TBLC in a PAP patient. A 49-year-old man complained of recurrent cough and tachypnea, and his symptoms did not fully resolve until the diagnosis was confirmed, and he was treated with whole lung lavage. Our patient was ultimately diagnosed with PAP by TBLC but not multiple tests for the bronchoalveolar lavage fluid (BALF). The patient was discharged quickly after whole lung lavage due to the fact that he did not develop pneumothorax under SBO. This case illustrates that TBLC is a supplementary examination for PAP, especially for those in whom BALF results fail to confirm a diagnosis. Moreover, our report highlights that SBO is necessary to effectively prevent pneumothorax during and after multiple TBLCs in PAP patients.

## 1 Introduction

Pulmonary alveolar proteinosis (PAP) is a diffuse lung disorder, mainly characterized by surfactant lipids and proteins in alveolar macrophages and alveoli due to defective clearance by alveolar macrophages, resulting in impaired gas exchange (1). Pathological diagnosis which is based on bronchoalveolar lavage (BAL) or biopsies is often required for diagnosing PAP (2). Compared with other methods of taking biopsies, transbronchial lung cryobiopsy (TBLC) has a higher diagnostic rate and manageable complications (3, 4). However, pneumothorax, one of the complications, occurred in 6–9.4% of patients after TBLC (4–6). A number of studies have shown that the application of various materials for occlusion after lung biopsy can reduce the risk of pneumothorax (7–9). Therefore, we attempted to perform SBO immediately at the end of TBLC to prevent the occurrence of pneumothorax.

There are some studies on PAP; however, there are no data available about selective bronchial occlusion (SBO) for the prevention of pneumothorax after TBLC in PAP patients. Here, we report such a rare case with a literature review.

## 2 Case report

A 49-year-old man was admitted to the Department of Respiratory and Critical Care Medicine due to recurrent cough and tachypnea for more than 1 year, and the relapse worsened for 3 months. At first, the patient was treated with cefprozil, dextromethorphan hydrobromide, and montelukast in other hospitals, but his symptoms did not improve. Subsequently, the patient went to several hospitals and had symptomatic treatment, the symptoms were slightly relieved, but the symptoms repeated immediately after the drugs were discontinued. Before 3 months of admission, his symptoms were getting progressively worse.

He was afebrile but tachypneic (respiratory rate 24 breaths per minute), with room air oxygen saturation of 87% and blood pressure of 104/53 mmHg. Breath sounds in both lungs were rough, with scattered moist rales in both lower lungs. He is a farmer and has a smoking history of more than 20 years.

A year ago, pulmonary computed tomography (CT) found interstitial inflammation in both lungs, but the IgM antibodies of respiratory pathogens were negative (respiratory syncytial virus, adenovirus, influenza virus, *chlamydia pneumoniae*, *legionella pneumophila*, *mycoplasma pneumoniae*, coxsackie virus, and enteric cytopathic human orphan virus). The pulmonary function test revealed that the percentage of forced vital capacity to the predicted value (FVC%), the percentage of forced expiratory volume in 1 second to the predicted value (FEV_1_%), FEV_1_/FVC, and diffusion capacity of lung carbon monoxide (DLCO) were 75.1, 74.1, 80.10, and 44.8%, respectively. The bronchoalveolar lavage fluid (BALF) was not milky in appearance, and the BALF cell analysis revealed *mycobacterium tuberculosis* DNA and non-*mycobacterium tuberculosis* were negative. At the same time, BALF samples were sent to an external clinical laboratory for metagenomic next-generation sequencing and pathological diagnosis. However, all pathogens of the number of sequences were low and had no diagnostic significance, pathological examination showed a few columnar epithelial cells and histiocytes, and periodic acid–Schiff (PAS) was negative. Moreover, the results of the patient’s two PAS by testing BALF samples at other hospitals prior to this admission were negative.

After this admission, initial investigations revealed normal renal and liver functions, D-dimer, coagulation function, and B-type natriuretic peptide and electrolyte. We further improved other examinations and found that anti-extracted nuclear antigens or antibodies, anti-cyclic citrullinated peptide, anti-neutrophil cytoplasmic antibodies, anti-O antibody, immunoglobulin E, and rheumatoid factor were negative. Preliminary consideration is interstitial pulmonary disease with infection. Therefore, he was given intravenous piperacillin–tazobactam (4.5 g each time, q8h) and methylprednisolone sodium succinate (40 mg, q12h). In addition, other treatments were used to improve symptoms.

**Figure 1:**
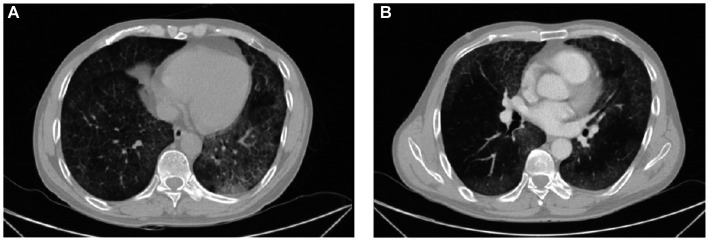
HRCT images. **(A,B)** Ground glass opacity and patchy shadow on bilateral lung, showing a crazy-paving pattern, mainly in the lower lobes.

Simultaneously, white cells, high sensitivity C-reactive protein, erythrocyte sedimentation rate, procalcitonin, and sputum examination were normal. The results of serum (1–3)-β-D-glucan and *Aspergillus* galactomannan were negative. However, arterial blood gas revealed a pH of 7.42 (7.35–7.45), PaCO_2_ 36 mmHg (35–45 mmHg), and PaO_2_ 63 mmHg (80–100 mmHg). Lactate dehydrogenase (LDH) and alpha-hydroxybutyrate dehydrogenase (α-HBDH) were 334.0 U/L (120–250 U/L) and 231 U/L (72–182 U/L), respectively. Lipid profile revealed total cholesterol and low-density lipoprotein were 6.74 mmol/L (<5.17 mmol/L) and 4.53 mmol/L (0.00–3.37 mmol/L), respectively. Color Doppler echocardiography showed mild aortic regurgitation, minimal mitral regurgitation, normal left ventricular systolic function, and decreased diastolic function. In the detection of tumor markers, serum cancer antigen 15-3(CA15-3), carcinoembryonic antigen (CEA), cytokeratin 19 fragment (CYFRA21-1), neuron-specific enolase (NSE), and progastrin-releasing peptide (ProGRP) were slightly increased, with 54.9 U/mL (0-30 U/mL), 8.94 ng/mL (0–4.5 ng/mL), 26.1 ng/mL (0–3.3 ng/mL), 24.6 ng/mL (0-18 ng/mL) and 73.8 pg./mL (0–67.42 pg./mL), respectively. Pulmonary high-resolution CT (HRCT) detected ground glass opacity and patchy shadow on bilateral lung, showing a crazy-paving pattern, mainly in the lower lobes (Figures 1A,B).

**Figure 2:**
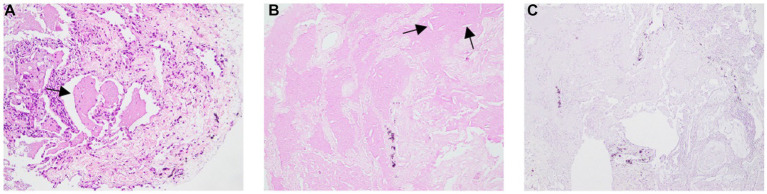
Pathological examination showing pink particles and needle-like fissures in alveolar dilation. **(B)** PAS was positive, **(C)** Congo red stain was negative.

Based on the clinical suspicion of PAP, we performed fiberoptic bronchoscopy again and collected BALF samples for examination, but the results were the same as a year ago. Considering a diagnosis of PAP, he received TBLC and SBO which consisted of tranexamic acid of 10 mL, thrombin of 500 IU, and cryoprecipitate of 2 U for the prevention of pneumothorax. He underwent mechanical ventilation under intravenous general anesthesia and electrocardiographic monitoring and a 50% fraction of inspired oxygen. Combined with the situation of chest CT, a balloon was placed in the outer basal segment of the right lower lobe, and a cryoprobe was placed in the anterior subsegment, posterior subsegment, outer basal segment, and posterior basal segment. After 5 s of freezing, four pieces of lung tissue with a size of approximately 3–8 mm were removed, and then the local area was squeezed by a balloon, and the plugging agent was injected in time. After squeezing for 2 min, the bronchoscope was pulled out after examination of the bronchus which showed there was no bleeding or other complications. Pathological examination showed pink particles and needle-like fissures in alveolar dilation, PAS was positive, and Congo red stain was negative (Figure 2).

We performed whole lung lavage immediately after the patient was clearly diagnosed. During the procedure, the cloudy milk liquid is irrigated until the collected liquid is clear. After that, the patient was given cefoxitin (2 g, q8h, ivgtt) to prevent infection and symptomatic treatment. Reexamination of pulmonary function showed that FVC%, FEV_1_%, FEV_1_/FVC, and DLCO were 95.2, 100.0, 85.38, and 42.3%, respectively. His symptoms improved significantly, and he was discharged from hospital 2 days later.

## 3 Discussion

In our case, a diagnosis of PAP was confirmed after TBLC. The patient mainly presented with recurrent cough and tachypnea, which gradually got worse. His symptoms did not fully resolve until the diagnosis was confirmed, and he was treated with whole lung lavage. His lung function also improved although diffusing capacity was not ameliorated. Obviously, a clear diagnosis is particularly important, but our patient was not diagnosed after multiple tests for BALF.

It is well known that PAP involves bronchioles and terminal bronchioles, excessive deposition of protein-like substances in alveoli and pulmonary interstitial fibrosis, which can lead to compensatory emphysema and subpleural bulla. Therefore, TBLC is more likely to lead to pneumothorax or even intractable pneumothorax in PAP patients. TBLC inherits a higher risk for pneumothorax than other bronchoscopic biopsy techniques (10). The patient had high-risk factors for pneumothorax, such as multiple previous TBLC procedures, a history of severe cough (11), large radiological fibrotic lesion (12), and undergoing mechanical ventilation under intravenous general anesthesia (13). A previous study suggested that SBO is an effective technique for treating intractable pneumothorax (14–16). SBO includes a plugging agent and plugging device, and the former can be dissolved and absorbed, but the latter needs to be removed after the cure of pneumothorax. Therefore, in order to prevent patients from developing pneumothorax during or after the operation of TBLC, a plugging agent was selected to seal the sampling site, with a significant effect. However, there are no previous studies on SBO for the prevention of pneumothorax after TBLC in a PAP patient. Our case report represents a chance to help fill the gap of knowledge relative to it.

It was reported that LDH is a predictor of the severity (17). Meanwhile, our patient’s α-HBDH level is high, which may be a potential indicator for the severity of PAP disease although no studies have reported it. Moreover, serum tumor markers such as CEA (18), CYFRA21-1 (19), and NSE (20) were also significantly associated with the severity of PAP. In our case, LDH, α-HBDH, CEA, CYFRA21-1, and NSE were only slightly increased, and all indicated a favorable prognosis. Although CA15-3 and ProGRP were also mildly elevated, there was no indication of gastrointestinal neoplasms, and the clinical significance of their rise in PAP patients is unclear.

There are several limitations to our study. We did not test for autoantibodies against granulocyte-macrophage colony-stimulating factor. Additionally, given conclusions were based on case reports, and multi-center and large-sample data are needed for verification in future.

In summary, TBLC is a supplementary examination for PAP, especially for those in whom BALF results fail to confirm a diagnosis. Furthermore, our report highlights that SBO is necessary to effectively prevent pneumothorax during and after TBLC in PAP patients.

## Data availability statement

The original contributions presented in the study are included in the article/supplementary material, further inquiries can be directed to the corresponding author.

## Ethics statement

The studies involving humans were approved by Zigong First People’s Hospital Ethics Committee. The studies were conducted in accordance with the local legislation and institutional requirements. The participants provided their written informed consent to participate in this study. Written informed consent was obtained from the individual(s) for the publication of any potentially identifiable images or data included in this article.

## Author contributions

H-MW: Data curation, Investigation, Methodology, Writing – original draft. Y-LW: Methodology, Resources, Supervision, Writing – review & editing. X-YH: Writing – original draft. Z-PD: Methodology, Resources, Supervision, Validation, Visualization, Writing – review & editing.
